# 

**Published:** 1903-03-28

**Authors:** 


### PUBLISHER'S NOTICE.

TO SUBSCRIBERS AND READERS OF "THE HOSPITAL."

### THE PRICE OF "THE HOSPITAL."

The circulation of The Hospital is now so large as to make it essential that the price shall approximate more closely to the actual cost of production than it has done in the past. It is therefore found necessary, after full consideration, to increase the price of this Journal from 2d. to 3d. per copy This change in price will take effect from the first number of Volume XXXIV., which will be issued on Saturday, April 4th, 1903. The terms of subscription from that date will be as follows:—

| | Great Britain. (Post free.) | Foreign and Colonial. (Post free.) |
|---|---|---|
| For one year ... ... | 15s. 0d. | 19s. 6d. |
| For six months ... ... | 7s. 6d. | 10s. 0d. |
| For three months ... | 3s. 9d. | 5s. 0d. |

| Monthly Parts. | Post free to any part of the World. |
|---|---|
| For one year ... ... ... | 15s. 6d. |
| For six months ... ... ... | 7s. 9d. |
| For three months ... ... ... | 4s. 0d. |

N.B.—Subscribers who have already paid their subscriptions will continue to receive The Hospital as issued at the old rates until such subscriptions expire.

**All new Subscriptions commencing on and after March 7, 1903, will be charged at the above Rates.**

Publishing and Editorial Offices, "The Hospital" Buildings, 28 & 29 Southampton Street, Strand, London, W.C.

### SCRAPS AND GLEANINGS.

Dr. Barnardo's Homes have been benefited to the extent of £34 by the efforts of the staff of the Ocean Accident and Guarantee Corporation, who arranged an evening entertainment amongst themselves and their friends on behalf of the homes.

A smoking concert in aid of the Home for Incurable Children, Maida Vale, was held on Thursday evening at the Crown Hotel, Aberdeen Place, N.W., Sir Samuel Scott, Bart., M.P., in the chair. Many artistes lent their services, and a feature of the evening was the powerful and dramatic recitation by Miss Lydia Lyons of an appropriate poem written specially for this occasion by Mr. Lincoln Springfield. Sales of copies of these lines in the room during the evening realised between £3 and £4 for the institution. As a result of the concert the Home's funds would benefit to the extent of £120.

### THE STUDENT OF MEDICINE.

### APPOINTMENTS.

**Victor George Alexander**, M.B., B.Ch., B.Sc.Edin., appointed to the District Surgeoncy of Standerton, South Africa. **M. H. Bland**, M.D.Glasg., C.M., appointed Medical Officer and Vaccinator of the Guisborough Union Workhouse. **W. C. Burton**, M.B., B.C.Edin., appointed Assistant Medical Officer of the Aston Union Workhouse. **A. J. Campbell**, M.R.C.S., L.R.C.P.Lond., appointed District and Workhouse Medical Officer of the Ross Union. **F. J. H. Coutts**, M.D.Vict., Ch.B., reappointed Medical Officer of Health for Blackpool. **E. F. N. Currey**, M.R.C.S.Eng., L.R.C.P.Lond., appointed District Surgeon Vereeniging, Transvaal. **R. A. Facey**, M.R.C.S.Eng., L.R.C.P.Lond., appointed District Medical Officer of the Ross Union. **G. B. Forge**, M.R.C.S., L.R.C.P.Lond., appointed Medical Officer of the Rochford Union. **Robert Algernon Fox**, M.B., M.S.Edin., appointed Visiting Medical Officer to the Rookwood and Newington Asylum for the Infirm, New South Wales. **H. Fraser**, M.D.Aberd., Ch.B., appointed Medical Officer to the Aberdeen Dispensary. **B. H. Herbert**, M.R.C.S.Eng., L.S.A., reappointed Medical Officer of Health to the Uttoxeter Urban District Council. **H. J. Hickin**, M.B., C.M.Glasg., L.M., appointed Government Medical Officer, Wei-Hai-Wee, North China. **G. Aubrey Jelly**, F.R.C.S.Edin., M.R.C.S.Eng., appointed Honorary Consulting Ophthalmic Surgeon to the Bury Infirmary. **M. R. Morrissey**, L.R.C.P., L.R.C.S.Edin., appointed Medical Officer for the Kill and Rathmore Dispensary District of the Nass Union. **John Murray**, M.B., C.M.Glasg., appointed Medical Officer of Health for the Llandrindod Wells Urban District. **H. J. Price**, F.R.C.S.Eng, L.R.C.P.Lond., appointed District and Workhouse Medical Officer of the Maldon Union. **R. B. Stamford**, F.R.C.S.Edin., L.R.C.P.Lond., M.R.C.S., appointed Honorary Medical Officer to the Loughborough and District Hospital and Dispensary. **E. Mansel Sympson**, M.A., M.D., B.C.Cantab., M.R.C.S., appointed Consulting Surgeon to the Lincoln General Dispensary. **James Templeton**, M.R.C.S., L.R.C.P.Lond., appointed Junior House Surgeon to the Bolton Infirmary. **J. G. Willis**, L.R.C.P., L.R.C.S.Edin., appointed Medical Officer of Health to the Bishop Auckland Rural District Council. **W. A. B. Young**, M.B., B.Ch.Vict., appointed Senior House-Surgeon to the Bolton Infirmary.

**"The Hospital" Institutional Library.**

"Hospitals and Asylums of the World." 4 Vols., with a Portfolio of Plans, complete £12 12s.

"Burdett's Hospitals and Charities, 1902." 5s.

"Burdett's Official Nursing Directory, 1903." 3s. net.

"Cottage Hospitals, General, Fever, and Convalescent." 10s. 6d.

"Uniform System of Accounts for Hospitals and Public Institutions." New Edition. 4s. net.

"Hospital Expenditure: The Commissariat." 2s. 6d.

"A Legal Handbook for the Use of Hospital Authorities." 2s. 6d.

"The Cottage Hospital Case Book or Register of Patients." 10s. and 12s. 6d.

"The Furnishing and Appliances of a Cottage." 6d.

All these are published by the Scientific Press, Ltd., and may be obtained through any bookseller, or direct from the publishers, 28 and 29 Southampton Street, Strand, London, W.C.

### PUBLISHER'S NOTICE.

TO SUBSCRIBERS AND READERS OF "THE HOSPITAL."

### THE PRICE OF "THE HOSPITAL."

The circulation of The Hospital is now so large as to make it essential that the price shall approximate more closely to the actual cost of production than it has done in the past. It is therefore found necessary, after full consideration, to increase the price of this Journal **from 2d. to 3d. per copy** This change in price will take effect from the first number of Volume XXXIV., which will be issued on Saturday, April 4th, 1903. The terms of subscription from that date will be as follows:—

| | Great Britain. (Post free.) | Foreign and Colonial. (Post free.) |
|---|---|---|
| For one year ... ... | 15s. 0d. | 19s. 6d. |
| For six months ... ... | 7s. 6d. | 10s. 0d. |
| For three months ... | 3s. 9d. | 5s. 0d. |

| Monthly Parts. | Post free to any part of the World. |
|---|---|
| For one year ... ... ... | 15s. 6d. |
| For six months ... ... ... | 7s. 9d. |
| For three months ... ... ... | 4s. 0d. |

N.B.—Subscribers who have already paid their subscriptions will continue to receive The Hospital as issued at the old rates until such subscriptions expire.

**All new Subscriptions commencing on and after March 7, 1903, will be charged at the above Rates.**

Publishing and Editorial Offices, "The Hospital" Buildings, 28 & 29 Southampton Street, Strand, London, W.C.

### STANDARD NURSING MANUALS.

### HANDBOOK FOR NURSES.

By J. K. Watson, M.D., M.B., C.M.

Second Edition. Crown 8vo. over. 400 pp, profusely illustrated, **5s.** net, **5s. 5d.** post free.

"A concise and comprehensive exposition of as much medical knowledge as a nurse needs. The work cannot but prove useful to those for whom it has been designed." *Scotsman.*

### MIDWIVES' POCKET BOOK.

**(With new chapter on the Midwives Bill).**

By Honnor Morten.

Small crown 8vo. cloth, **1s.** post free.

An exceedingly useful handbook for those entering for the L.O.S. certificate as well as for qualified Midwives.

### NURSING: ITS THEORY AND PRACTICE.

By Percy G. Lewis, M.D., M.R.C.S., L.S.A.

New and Enlarged Edition.

Crown 8vo. over 400 pp. profusely illustrated, **3s. 6d.** post free.

"Nurses will find the book full of interest and instruction, which cannot fail to assist them in their work." *British Medical Journal.*

### ELEMENTARY ANATOMY AND SURGERY FOR NURSES.

By William McAdam Eccles, M.S.Lond., M.B., &c.

Crown 8vo. Illustrated, cloth, **2s. 6d.** post free.

"Valuable to nurses who are beginning the study of anatomy. The instruction is given very clearly and concisely, and the reader is able to glean much information in a very condensed form."—*Nursing Notes.*

### NURSING: ITS PRINCIPLES AND PRACTICE.

By Isabel Adams Hampton.

New and Enlarged Edition.

Second Edition. Crown 8vo. illustrated, **7s. 6d.** net, post free **7s. 10d.**

"The subject has been scientifically, carefully, and completely treated by Miss Hampton."—*The Hospital.*

### ELEMENTARY PHYSIOLOGY FOR NURSES.

By C. F. Marshall, M.D., B.Sc., F.R.C.S.

Crown 8vo. Illustrated, cloth, **2s.** post free.

"Nurses will find in it all that they require to know of the subjects treated."—*Daily Chronicle.*

### OPHTHALMIC NURSING.

By Sydney Stephenson, M.B., F.R.C.S.E.

Second Edition. Crown 8vo. illustrated, with glossary of terms, cloth, **3s. 6d.** net, **3s. 10d.** post free.

"May very advantageously be studied by nurses who are about to enter the Ophthalmic service of a hospital." *Lancet.*

### ART OF MASSAGE.

By A. Creighton Hale.

Second Edition. Demy 8vo. Profusely illustrated, cloth gilt, **6s.** post free.

"Mrs. Hale seems to have had considerable experience, not only as a practitioner, but as a teacher of the art, and explains in a series of clearly illustrated articles the many complaints for which massage has been found beneficial, and the special manipulations required in each case." *Morning Post.*

### NURSING IN DISEASES OF THE THROAT, NOSE AND EAR.

By P. Macleod Yearsley, F.R.C.S.Eng., M.R.C.S., L.R.C.P.Lond.

Demy 8vo. Illustrated, cloth, **2s. 6d.** post free.

"Of practical service to every nurse who wishes to be thoroughly trained." *Scottish Medical and Surgical Journal.*

### ART OF FEEDING THE INVALID.

By a Medical Practitioner and a Lady Professor of Cookery.

New and Popular Edition. Demy 8vo. cloth, **1s. 6d.** post free.

"A new edition of this valuable work is very welcome, and we heartily commend it to all who desire reliable and practical instruction in sick cookery."—*Nursing Notes.*

### SURGICAL WARD-WORK AND NURSING.

By Alexander Miles, M.D.Edin., C.M., F.R.C.S.E.

Revised Edition. Demy 8vo. Illustrated, cloth gilt, **3s. 6d.** net, **3s. 10d.** post free.

"An illustrated manual which will furnish much needed guidance to the student and the nurse in the early stages of their apprenticeship."—*The Times.*

### HOSPITAL SISTERS AND THEIR DUTIES.

By Eva C. E. Lückes, Matron to the London Hospital.

Third Edition. Crown 8vo. Cloth gilt, **2s. 6d.** post free.

"An admirable guide to the beginner anxious to take up a Sister's work. Its pages bring help and knowledge imparted in a very sympathetic, straightforward manner by one who has had practical experience in the lesson she teaches."—*Gentlewoman.*

*COMPLETE CATALOGUE POST FREE ON APPLICATION.*

The SCIENTIFIC PRESS, LTD., 28 & 29 Southampton Street, Strand, London, W.C.

